# Impact of cryopreservation agents on sperm quality, DNA fragmentation, and apoptotic markers in fertile and infertile males

**DOI:** 10.1038/s41598-025-15456-0

**Published:** 2025-08-17

**Authors:** Amoura M. Abou-El-Naga, Mohamed E. Abdraboh, Mohammed A. El‑Magd, Sameh Mansour, Hend Abd El-Halim Mansour

**Affiliations:** 1 Zoology Department, Faculty of Science, Mansoura University, Mansoura, Egypt; 2 Anatomy Department, Faculty of Veterinary Medicine, Kafrelsheikh University, Kafr El-Shaikh, Egypt; 3 Zoology and Entomology Department, Faculty of Science, Al-Azhar University, Cairo, Egypt

**Keywords:** Semen cryopreservation, Male fertility, Artificial insemination (AI), Intra-cytoplasmic sperm injection (ICSI), The DNA fragmentation, Sperm chromatin structure assay (SCSA), Health occupations, Medical research

## Abstract

Semen cryopreservation is a crucial technique for preserving male fertility, playing a vital role in assisted reproductive procedures by storing frozen semen samples for artificial insemination (AI) and intra-cytoplasmic sperm injection (ICSI) to enhance reproductive success rates. This study aims to identify the most effective cryopreservation methods and assess their impact on semen quality, particularly sperm DNA fragmentation. A total of 30 semen samples were categorized into fertile and infertile groups. DNA fragmentation analysis was conducted using the Sperm Chromatin Structure Assay (SCSA). Each sample was divided into three portions and frozen using different cryoprotectants: (egg-yolk + glycerol), (sucrose + glycerol), and (glycerol alone). After one month of storage, the samples were analyzed to determine the most effective medium. The findings revealed a decline in sperm motility post-freezing compared to fresh samples, along with a slight increase in morphological abnormalities. Additionally, there was a rise in sperm DNA fragmentation and an increase in apoptotic marker (Caspase-3) levels after the freezing process. The study concluded that cryopreservation and thawing caused some degree of sperm cell damage, with infertile samples being more adversely affected than fertile ones.

## Introduction

Cryopreservation is frequently used in ARTs to preserve fertility in some important instances, such as chemotherapy, radiation therapy, cancer, and surgical procedures that may cause testicular injury or ejaculatory failure^1^. In cases of oligozoospermia, where more than one sample must be collected and created for Intrauterine Insemination (IUI), In Vitro Fertilisation (IVF), and Intracytoplasmic Sperm Injection, the male partner in assisted reproductive systems greatly benefits from cryopreservation of semen and this technique^2,3^. According to **Mansour et al.**^4^semen cryopreservation is not just for human use but also for the preservation of endangered species’ diverse genetic makeup. Although cryopreservation has a beneficial role, it can also cause detrimental changes to sperm composition and functions. Sperm cells are less susceptible to damage from cryopreservation than other cell types because of their high membrane fluidity and low water content (approximately 50%)^5^. Cryoprotectant, also known as cryoprotective agent, is used to keep human sperm from getting damaged during cryopreservation. But adding cryoprotectant during the freezing process and taking it out during the thawing process reduces the fertility of human semen by destroying sperm cell membranes and decreasing sperm motility^6^. The penetrating sperm cell body that occurs when a cryoprotective substance enters a sperm cell prior to freezing and when it is removed after thawing causes osmotic injury, which damages the sperm cell during the cryopreservation process^7^. Both human and animal sperm cryopreservation uses glycerol as a cryoprotectant. These cryoprotectants are hydrophilic enough to mix readily with water and hydrophobic enough to quickly penetrate cell membranes^8^. Glycerol, a cryoprotectant that shields sperm cells from osmotic damage, is frequently enlarged using cryobuffers made of non-permeating macromolecules, such as egg yolk or citrate^9^. The three main components of sperm validity count, motility, and morphology are measured by semen analysis; these parameters can be used to characterise the condition of fertility cases and sperm vitality^10^. Depending on the type of defect, a decrease in the overall capacity for fertilisation may result from faulty human sperm cells. Additionally, aberrant sperm DNA may be present. Sperm membrane shape and function changes during freezing-thawing processes may result in damage to DNA sperm cells, which lowers frozen semen fertility^11^. DNA fragmentation may be connected to these anomalies. With the exception of other mammalian sperm cells, the degree of condensation of the chromatin structure of human sperm varies greatly within a single ejaculate. This discrepancy could result from varying degrees of disulphide bonding and histone replacement in each sperm throughout the spermatogenesis process. As a result, certain ejaculates’ sperm have been shown to have an incomplete condensation process. In human sperm cells, the process of DNA condensation is not finished in the testis during sperm production. Instead, it is finished when sperm pass through the epididymis, where thiol groups (R-SH) oxidise and form a large number of disulphide bonds, which results in more stable sperm nuclei. Following ejaculation, zinc penetrates the chromatin of sperm and interacts with free thiol groups to stabilise its quarternary shape^12^. Sperm cells contain two forms of defence mechanisms against oxidative stress on their DNA: condensation of sperm DNA and seminal plasma. This is because the sperm cells lose much of their cytoplasm at the last stage of spermatogenesis, which also causes them to lose most of their cytoplasmic defense enzymes. Both the fertile and infertile groups experience significant damage to their sperm chromatin condensation, sperm morphology, and sperm cell membrane integrity following the freezing-thawing process; the fertile group’s normal semen sample resists the damage better than the infertile group’s low semen values. Additionally, sperm chromatin condensation degrades much more in the infertile group than in the fertile group. As with other antioxidants that impede oxidation-reduction interactions, oxidative stress can be triggered by an increase in ROS generation and the activity of antioxidants such as glutathione peroxidase, superoxide dismutase, and catalase in semen plasma^13^. The single strand of sperm DNA is really damaged by oxidative stress, but alterations in protein and lipid peroxidation are also possible. Caspases have a role in the processes of sperm cell DNA fragmentation and death. DNA fragmentation higher than normal controls indicates the presence of active caspase-3 in sperm cells. Additionally, low-motility sperms had higher amounts of apoptotic markers than high-motility sperms^14^. Individuals whose DNA fragmentation surpasses 20% should have their rates evaluated multiple times and at various points in time. These studies generally point to the following conclusions: a percentage of DNA fragmentation below 15% should be regarded as normal, a percentage between 15% and 30% has some issues with fertility, and males with a percentage above 30% have significant difficulties conceiving naturally^15^.

This study aimed to evaluate the impact of different cryoprotectants on sperm cells to determine which formulation most effectively preserved sperm characteristics and to assess the detrimental effects of cryopreservation on sperm DNA integrity in both fertile and infertile men.

## Materials and methods

### Experimental groups and sperm cryopreservation and thawing

A total of thirty semen samples were taken from male humans aged 29 to 41. Following three to five days of sexual abstinence, semen samples were obtained via masturbation. The total number of samples was split into two groups:

A. Fertile group: Fifteen healthy normal donors were selected on the basis of a normal semen analysis according to the World Health Organization (WHO) classification^16^.
B. Infertile group: Fifteen unhealthy samples of smokers donors comprise the second group.

Each group subdivided into three groups underwent cryopreservation using three distinct cryoprotective media:

1. Sperm freezing media including egg yolk and glycerol^17^.

(2) Glycerol and sucrose-containing sperm freezing media^18^.

(3) Glycerol-only sperm freezing media^7^.

All these subgroups compared to the fresh sample. Then samples put in liquid nitrogen at −196 degrees Celsius, sperm freezing medium glycerol vitrife from Sweden, sperm freezing medium egg-yolk with glycerol Irvine from the USA, and sperm freezing medium sucrose with glycerol fertiliser from Belgium. WHO^16^ states that in order to manually freeze semen, one millilitre of cryoprotective freezing media should be added drop by drop to one millilitre of semen sample at each cryovial step, and the mixture should be thoroughly mixed. After that, the cryovial was placed in a freezer at −20 °C for 30 min, then in a liquid nitrogen vaporiser at −80 °C for 10 to 15 min, and finally, at −196 °C for storage. Semen samples were put in a rack and allowed to get to room temperature, which took ten minutes.

### Semen analysis (microscopic examination)

**Sperm count**: repared a dilution solution and combined it with a fixative-treated semen sample. Using a hemocytometer chamber, 10 microns of semen were added to each chamber and placed in a humid chamber. The hemocytometer includes nine 1 mm x 1 mm grids. The fifth central grid, which has 25 large squares and 16 small squares each, is used to count sperm cells. Using an X400 magnification microscope with an X10 ocular lens and an X40 objective lens, at least 200 sperm cells were counted in the central grid. Calculated sperm cells count by following equation.

$${\rm Sperm\: cells\: count = N/20n \times D.F. = ....... X\: 10^6/ml}$$

When; N is the no. of sperm cells which was counted, D.F. = the dilution factor, n = the no. of rows in central grid, b = the volume of semen in each row in central grid.

**Sperm motility**: Before removing a drop for the motility test, the semen sample was thoroughly mixed. The semen sample drop was placed on the slide glass and allowed to wander for one minute. Using an X400 magnification, count at least 200 sperm in each of the five fields on the slide. Provide the percentages for each of the three types of motility: immotility (IM), non-progressive motility (NP), and progressive motility (PR). Sum the PR and NP to get the overall motility.

**Sperm morphology**: Semen smears were prepared on a microscopic slide. The semen sample was thoroughly mixed, and a 5–10 µl aliquot was taken depending on its concentration. If the concentration was below 2 × 10⁶ sperm/ml, the sample was centrifuged at 600 g for 10 min, after which most of the supernatant was removed. The remaining pellet was mixed with the residual supernatant. A drop of this prepared sample was placed on a microscopic slide, and another slide was used to spread it evenly across the surface. The smear was left to air dry before being fixed with ethanol for cell dehydration. Gradual rehydration with different ethanol concentrations was performed to facilitate haematoxylin staining, followed by distilled water to further prepare the smear. Haematoxylin was applied to stain the sperm nucleus blue, after which ethanol was used to dehydrate the smear for orange G staining. Orange G was then applied to stain the cytoplasm pink. For sperm morphology assessment, the slide was examined under a ×100 oil-immersion bright-field objective with a ×10 eyepiece, ensuring clearer imaging. A minimum of 100 sperm were counted to determine the percentage of normal and abnormal sperm.

### Determination of semen values by computer-assisted sperm analyzing (CASA) system

According to Jasko et al.^19^computer-assisted sperm analysis (CASA) is also utilised to ascertain the count, motility, and morphology of semen samples in order to guarantee the correct values.

### Determination of DNA fragmentation index (DFI) of sperms

The DNA Fragmentation Index (DFI) was assessed using the Sperm Chromatin Structure Assay (SCSA)^20^. Double-distilled water (ddH₂O) was used to prepare all solutions, which were stored at 4 °C for several months. To determine DNA fragmentation, the following solutions were prepared: (1) Acridine Orange (AO) Stock Solution 50 mg of AO was dissolved in ddH₂O and filtered using filter paper. (2) Acid-Detergent Solution A mixture of 20 ml of 2.0 N HCl, 4.39 g NaCl, and 0.5 ml Triton X-100 was dissolved in ddH₂O to a final volume of 500 ml. (3) Citric Acid Buffer (0.1 M) Prepared by dissolving 21.01 g of citric acid in 1.0 L of ddH₂O. (4) Sodium Phosphate (Na₂PO₄) Buffer (0.2 M) Prepared by dissolving 28.4 g of Na₂PO₄ in 1.0 L of ddH₂O. (5) Staining Buffer (pH 6.0) Prepared by combining 370 ml of 0.1 M citric acid, 630 ml of 0.2 M Na₂PO₄, 372 mg EDTA, and 8.77 g NaCl, followed by thorough mixing.

The AO working solution was prepared by adding 600 µl of AO stock solution to 100 ml of staining buffer and stored at 4 °C for up to two weeks. For analysis, the semen sample was diluted with Phosphate Buffered Saline (PBS) containing 2.0 mM MgCl₂ until reaching a concentration of 1–2 million cells/ml. A 200 µl aliquot of the diluted sample was placed in a small test tube, followed by the addition of 400 µl of acid-detergent solution. After 30 s, 1.2 ml of AO working solution was added and mixed thoroughly. After 3 min, the sample was analyzed using a flow cytometer, recording data for 5000 cells. The procedure was repeated at least twice to ensure accuracy. The DNA Fragmentation Index (DFI%) for each sample was then determined using the SCSA method.

### RNA extraction from sperms

Pure RNA was extracted using a total RNA purification kit following the manufacturer’s protocol. After thawing, the semen samples were subjected to a two-step washing process using phosphate-buffered saline (PBS) and centrifugation at 500 × g for 10 min to help remove egg yolk components. The supernatant was carefully discarded, and the sperm pellet was resuspended and washed again to ensure the removal of residual egg yolk. Briefly, samples were homogenized in Lysis Buffer containing guanidine thiocyanate, a chaotropic agent that protects RNA from endogenous RNases. The lysate was then mixed with ethanol and loaded onto a purification column. The combination of ethanol and the chaotropic salt facilitated RNA binding to the silica membrane while the lysate was spun through the column. Impurities were effectively removed by washing the column with specific wash buffers. Finally, pure RNA was eluted under low ionic strength conditions using nuclease-free water. For complementary DNA (cDNA) synthesis, RevertAid H Minus Reverse Transcriptase, a genetically modified M-MuLV RT, was used to convert RNA into cDNA. To prepare the reaction, 5 µg of template RNA was placed in a sterile, nuclease-free tube on ice, followed by the addition of 0.5 µg Oligo dT. The volume was adjusted to 12.5 µl using DEPC-treated water. Next, 4 µl of 5× Reaction Buffer, 0.5 µg of RiboLock RNase Inhibitor, 2 µl of dNTP Mix, and 1 µl of RevertAid™ H Minus Reverse Transcriptase were added to the tube. After gentle mixing, the reaction mixture was incubated at 42 °C for 60 min, followed by termination of the reaction at 70 °C for 10 min.

### Quantification of RNA using nano drop

To ensure the RNA and cDNA concentrations were sufficiently pure for real-time PCR, their quantities were measured. For highly pure samples free from contaminants such as organic solvents, nucleic acids, phenol, carbohydrates, and proteins UV light absorption by the ring structures of purines and pyrimidines was used to determine nucleic acid concentration. The Q5000 spectrophotometer automatically performed all necessary measurements and calculations. First, the instrument’s upper arm was lifted, and 1.5 µl of blank buffer was placed on the lower surface. The upper arm was then closed, and the “Measure” button was pressed to obtain the blank reading. After measurement, the upper arm was lifted, and any remaining blank buffer was carefully wiped from both surfaces using soft, dry paper. For pure RNA, the OD260/OD280 ratio should be ≥ 2. A lower ratio indicates contamination, typically from proteins (which absorb at 280 nm) or phenol.

### Determine caspase-3 expression by real time PCR

The expression of caspase-3 mRNA in sperm was analyzed using real-time PCR with SYBR Green and GAPDH as a reference gene. Specific primers were designed based on published human sequences using Primer3 software. To ensure primer specificity, sequence similarity was checked using BLAST, confirming that the designed primers were unique. Following the manufacturer’s instructions, lyophilized primers stored at −20 °C were brought to room temperature before use. The equilibrated primers were briefly spun down for 3 s using a spin-centrifuge vortex. Both forward and reverse primers were then diluted with RNase-free water to prepare a 100 µM stock solution, followed by gentle inversion for 2 min at room temperature. The stock primers were further diluted with RNase-free buffer (pH 8.0) to obtain a 5 µM working solution, which was stored at −20 °C until use as shown in Table 1.

**Table 1:** Forward and reverse primers sequence for candidate genes:.

Gene	Forward primer (^/^5 ------ ^/^3)	Reverse primer (^/^5 ------ ^/^3)	Accession number
Caspase3	TTAATAAAGGTATCCATGGAGAACACT	TTAGTGATAAAAA TAGAGTTCTTTTGTGAG	NM_032991.2
GAPDH	TGCACCACCAACTGCTTAGC	GGCATGGACTGTGGTCATGAG	NM_002046

### Relative expression

The polymerase chain reaction (PCR) mixture was prepared in a 25 µl reaction volume, containing: 3 µl of cDNA template (10–20 ng/µl), 12.5 µl of 2X Maxima SYBR Green/ROX qPCR Master Mix, 1 µl of forward primer (10 µM), 1 µl of reverse primer (0.1–0.5 µM) and 7.5 µl of nuclease-free water. The reaction mixture was placed in a Step One Plus real-time thermal cycler (Applied Biosystems, Life Technologies, USA) and subjected to the appropriate PCR conditions. At the end of the cycles, a melt curve analysis was performed by gradually increasing the temperature from 60 °C to 95 °C. For gene expression analysis, the housekeeping gene GAPDH was used as an internal reference. The relative expression of the target gene was determined using the 2^-∆∆Ct method, where the Ct (threshold cycle) values of the target gene were normalized to those of GAPDH.

### Statistical analysis

The means ± S.E. were used to express all the data. Duncan’s multiple range test (DMRT) was used to generate the individual comparisons, and two-way analysis of variance (ANOVA) was used to assess the statistical significance using SPSS, 18.0 software, 2011. When *p* < 0.05, values were deemed statistically significant.

## Results

### Semen analysis results

The validity of sperm cells is detected by counting, analysing, and morphing sperm cells in semen samples from two groups fertile and infertile, both before and after cryopreservation. This is done using computer-assisted sperm analysis (CASA) and the manual method of semen analysis. All semen samples showed changes in sperm cell motility and morphology following freezing-thawing procedures, although the count remained same.

**Sperm motility**: Total motility and progressive motility of sperm cells was determined in the two groups of fertile and infertile semen samples before and after cryopreservation.

**Fig. 1:**
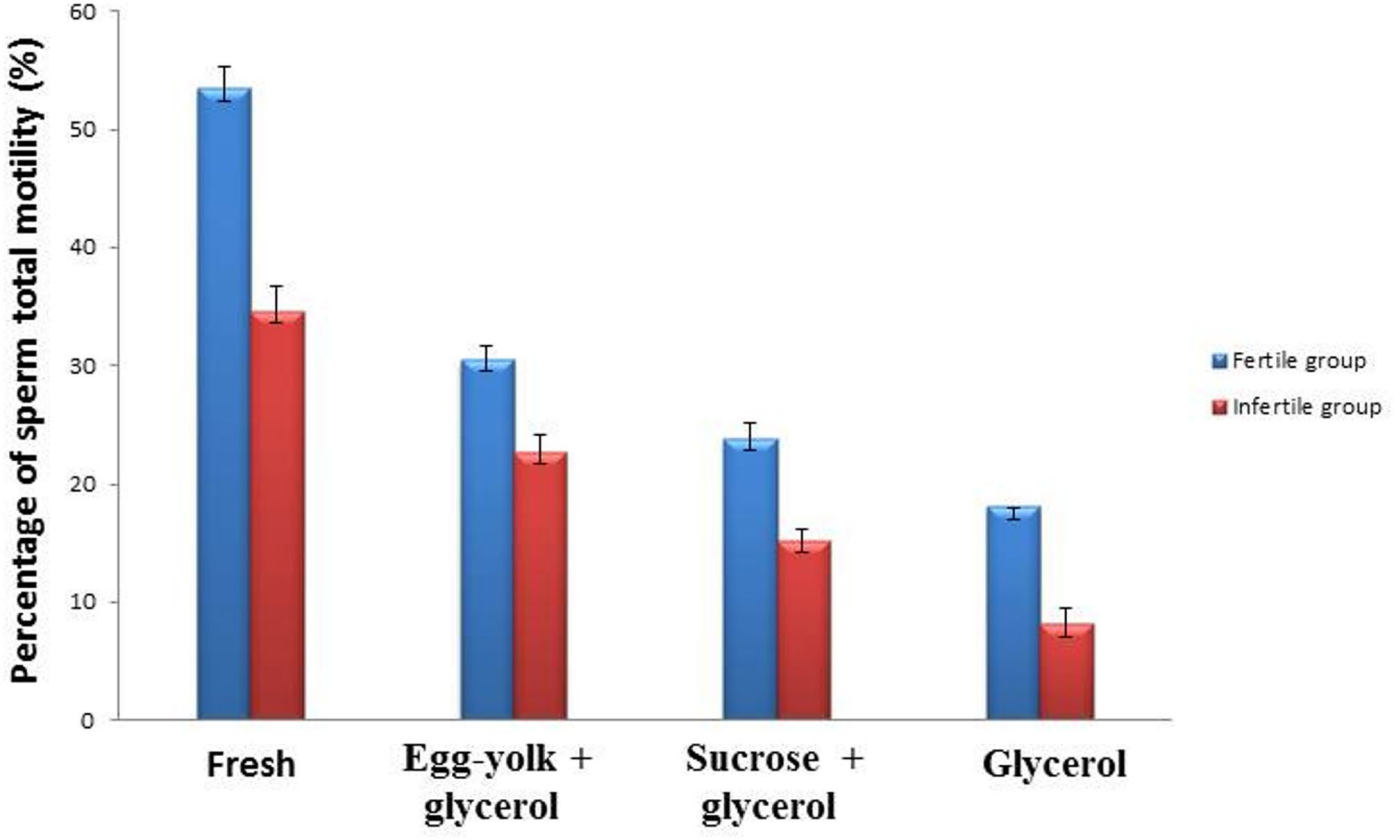
Graphical presentation of semen analysis of the sperm total motility in semen samples in the fertile and infertile groups. Data was expressed as mean ± SEM when (*n* = 15) in triplicate in each group.

The obtained results showed a considerable (*P* ≤ 0.05) decrease of the sperm total motility in fresh and frozen semen samples of fertile and infertile groups. The data showed in the egg-yolk + glycerol group (30.49 ± 1.21; 22.69 ± 1.44), sucrose + glycerol group (23.82 ± 1.34; 15.21 ± 1.04) and in glycerol group (18.05 ± 1.46; 8.09 ± 1.45) as compared to fresh semen group (53.45 ± 1.79; 34.57 ± 2.09) as represented in Fig. (1).

**Fig. 2:**
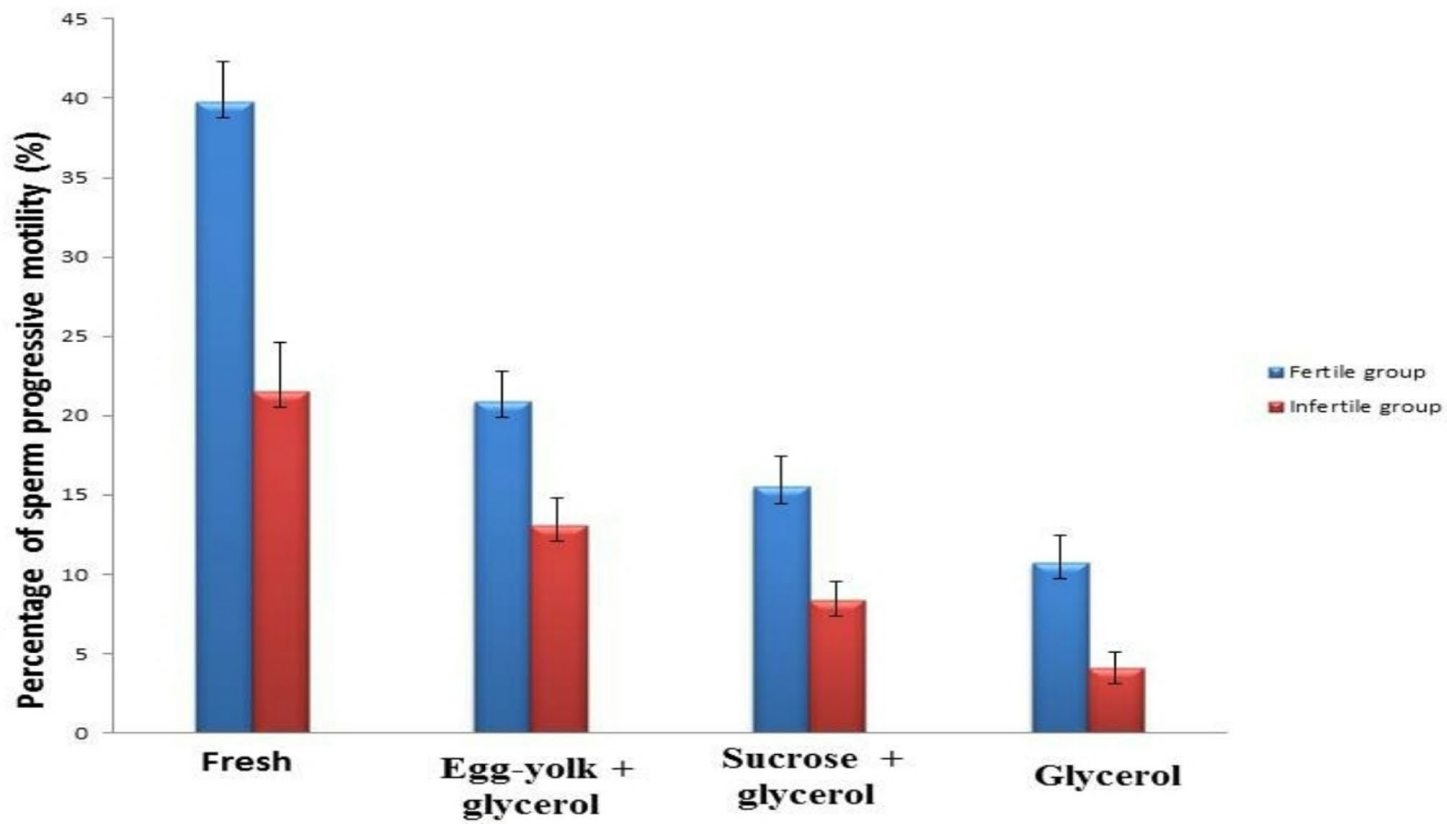
Graphical presentation of semen analysis of sperm progressive motility in semen samples in the fertile and infertile groups. Data was expressed as mean ± SEM when (*n* = 15) in triplicate in each group.

The obtained results showed a considerable (*P* ≤ 0.05) up-regulation in the expression of the sperm progressive motility in fresh and frozen semen samples of fertile and infertile groups. The data showed in the egg-yolk + glycerol group (20.87 ± 1.93; 13.08 ± 1.76), sucrose + glycerol group (15.50 ± 1.98; 8.39 ± 1.15) and in glycerol group (10.74 ± 1.68; 4.13 ± 1.01) as compared to fresh semen (39.76 ± 2.54; 21.55 ± 3.10) as represented in Fig. (2).

**Sperm morphology**: The morphology of sperm cells was determined in the two groups of fertile and infertile semen samples before and after cryopreservation. Data was expressed as mean ± SEM when *n* = 15 in triplicate in each group.

**Fig. 3:**
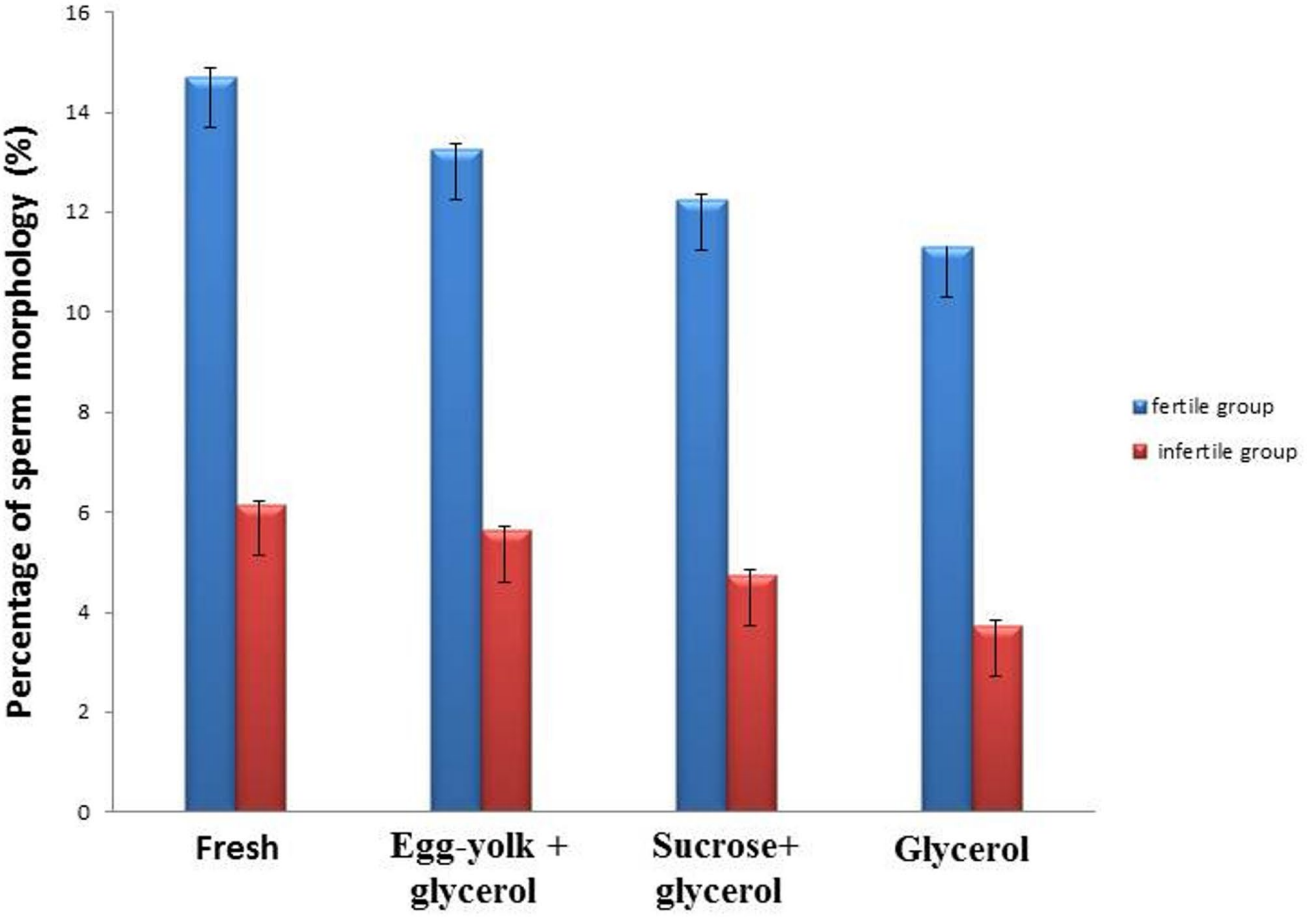
Graphical presentation of semen analysis of the sperm morphology in semen samples in the fertile and infertile groups. Data was expressed as mean ± SEM when (*n* = 15) in triplicate in each group.

**Fig. 4:**
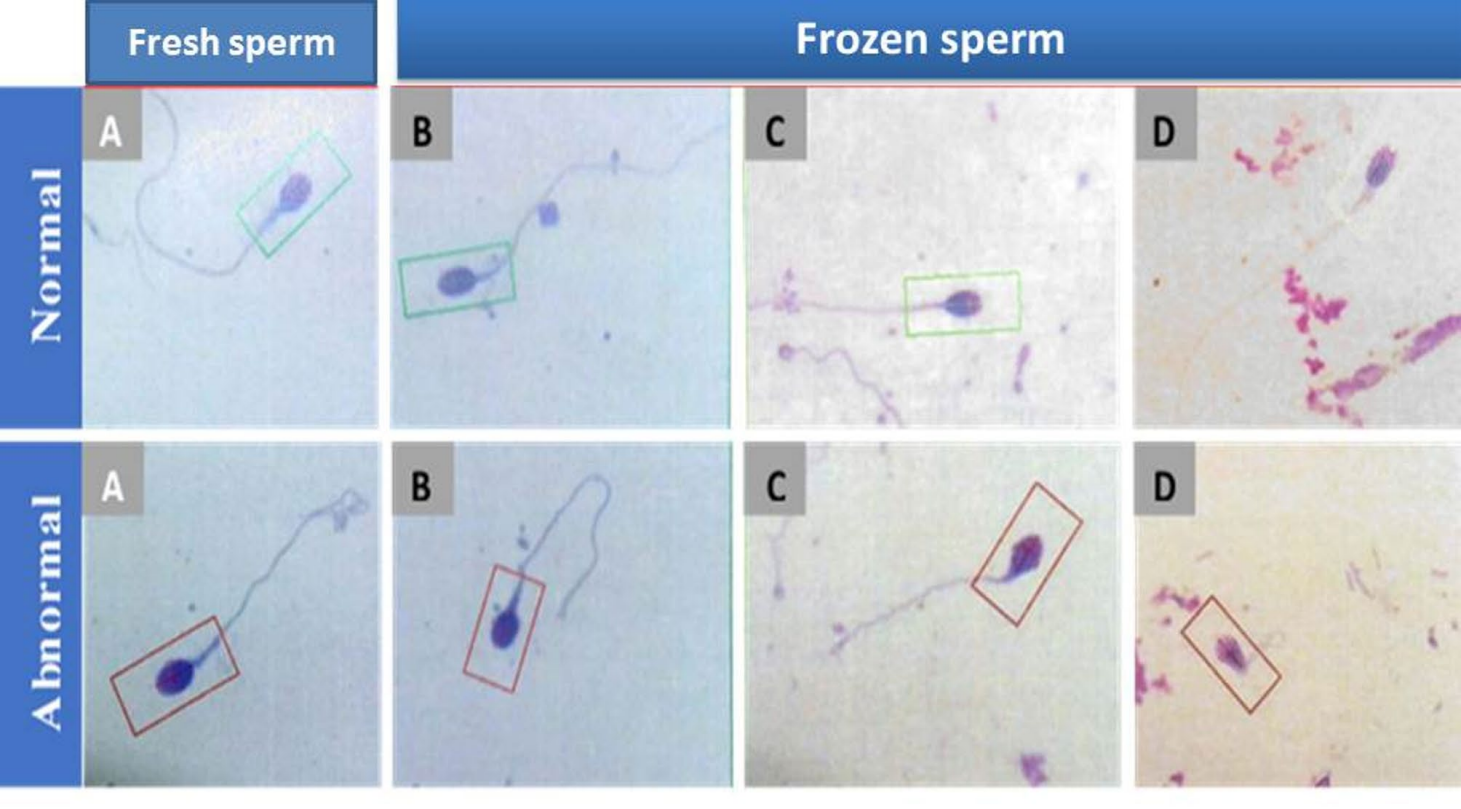
Normal forms and abnormal forms in fresh and frozen samples in the fertile group. Data was expressed as mean ± SEM when (*n* = 15) in triplicate in each group.

**Fig. 5:**
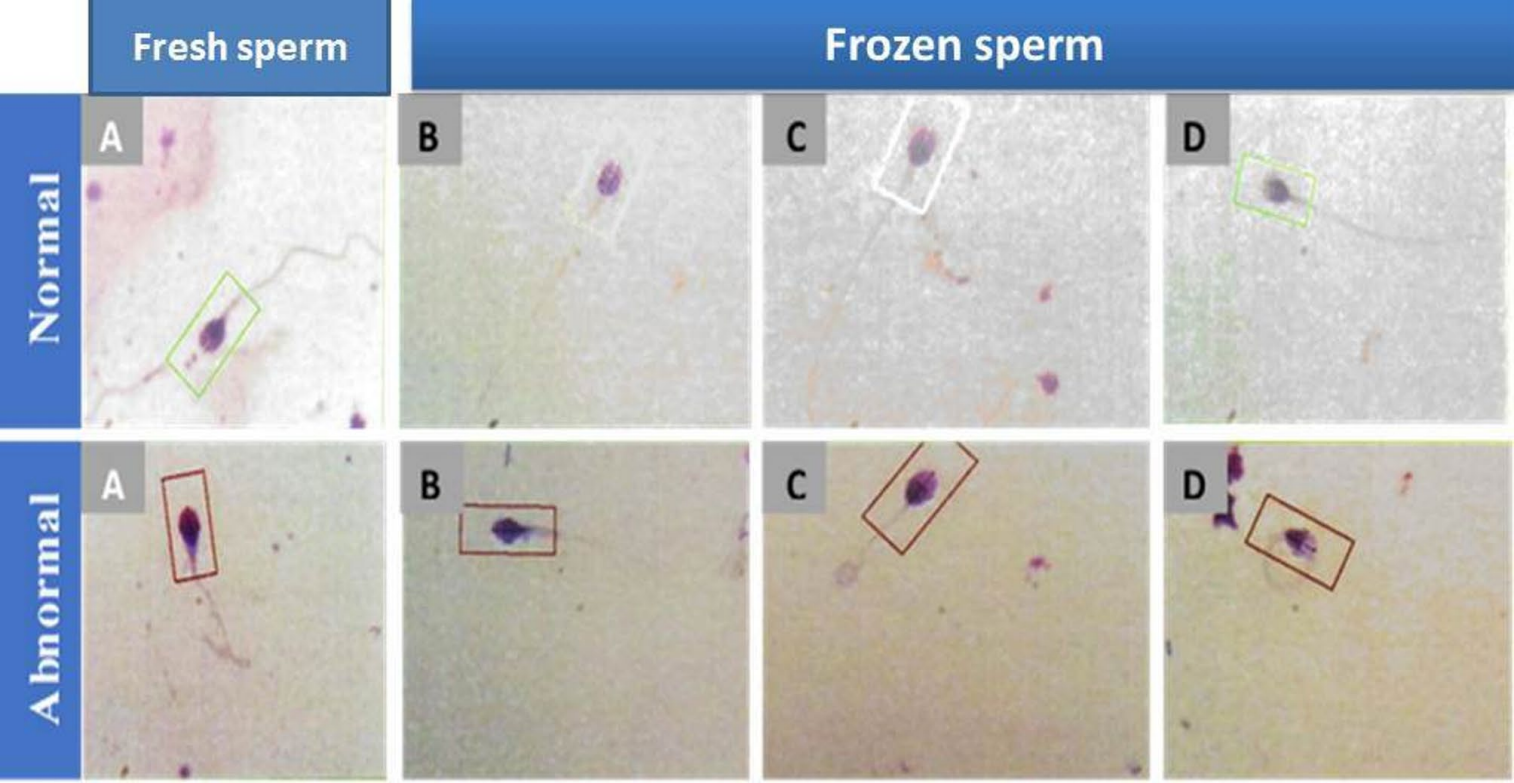
Normal forms and abnormal forms in fresh and frozen samples in the infertile group. Data was expressed as mean ± SEM when (*n* = 15) in triplicate in each group.

-The current results showed a considerable (*P* ≤ 0.05) decline of the sperm morphology in fresh and frozen semen samples of fertile and infertile groups. The result showed (13.27 ± 0.12; 5.62 ± 0.12) in egg-yolk + glycerol group, (12.25 ± 0.11; 4.75 ± 0.11) in sucrose + glycerol group and (11.32 ± 0.11; 3.72 ± 0.13) in the glycerol group compared to fresh semen group(14.70 ± 0.18; 6.13 ± 0.09) as shown in Figs. (3, 4, 5)

### Effect of cryoprotective agents on sperm DNA fragmentation (SDF) in semen samples of fertile and infertile group groups

**Fig. 6:**
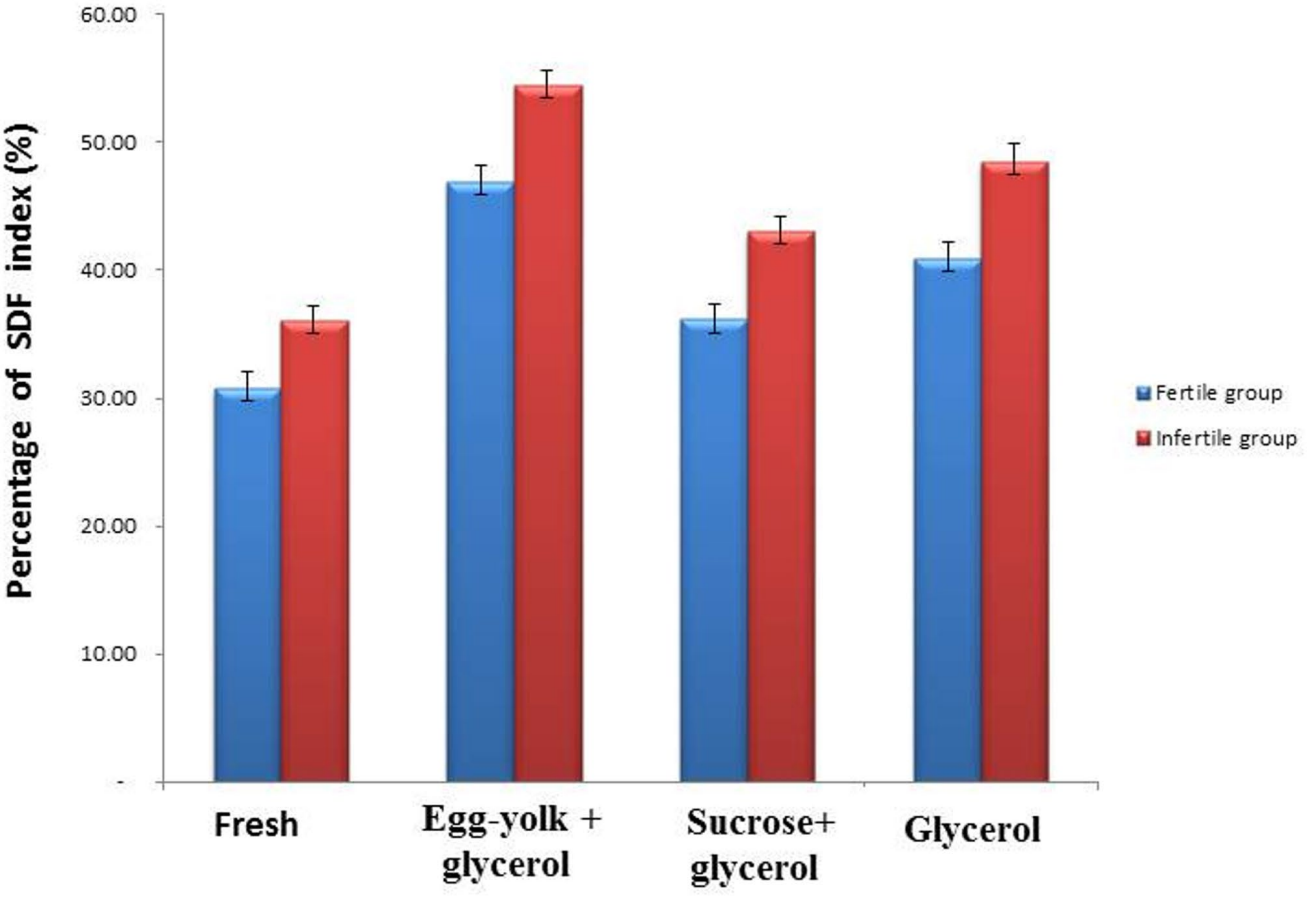
Graphical presentation of analysis of the value of sperm DNA fragmentation (SDF) in semen samples in the fertile and infertile groups. Data was expressed as mean ± SEM when (*n* = 15) in triplicate in each group.

**Fig. 7:**
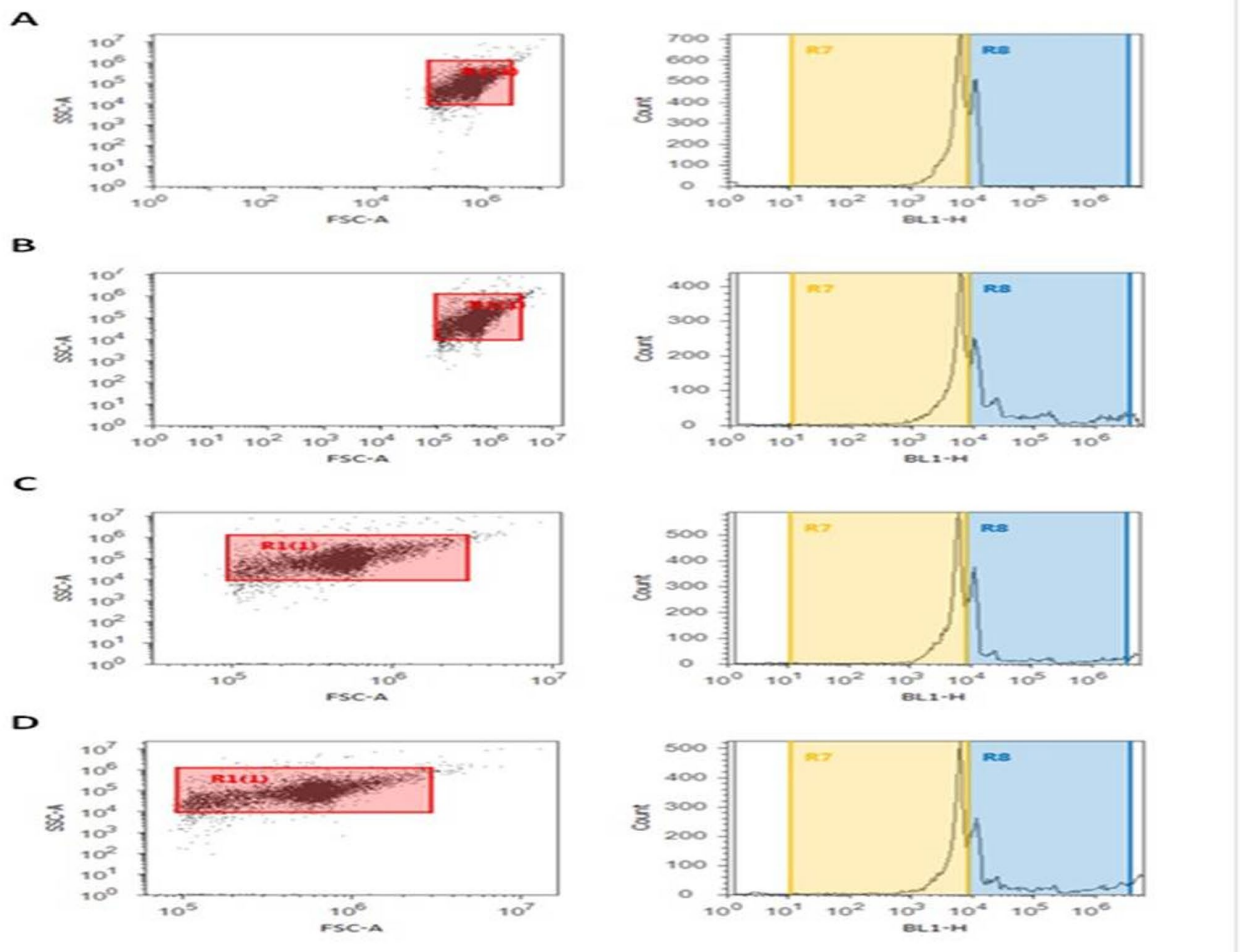
Sperm DNA fragmentation (SDF) of fresh and frozen semen samples in the fertile group. Data was expressed as mean ± SEM when (*n* = 15) in triplicate in each group.

**Fig. 8:**
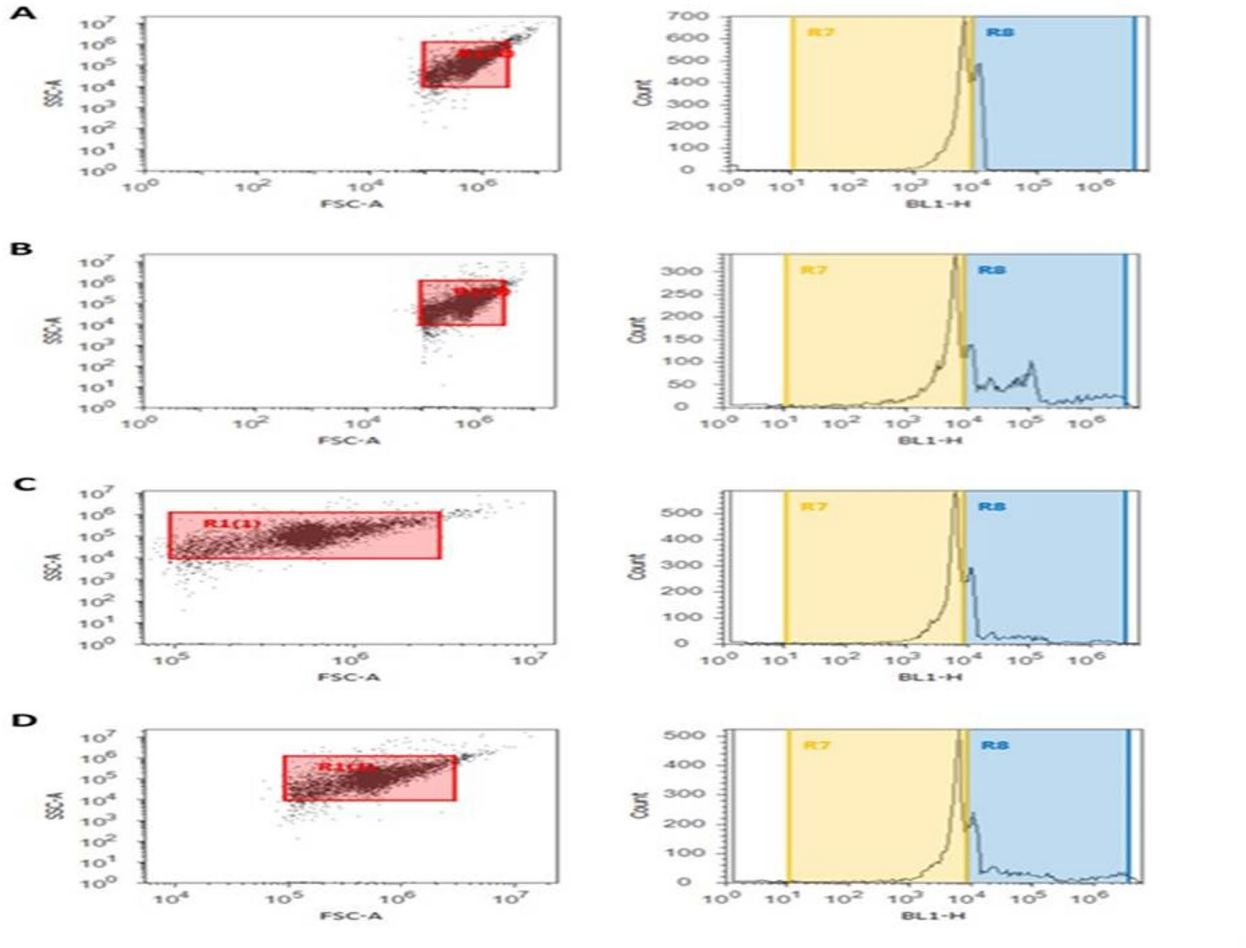
Sperm DNA fragmentation (SDF) of fresh and frozen semen samples in the infertile group. Data was expressed as mean ± SEM when (*n* = 15) in triplicate in each group.

The obtained results showed a considerable (*P* ≤ 0.05) increase of the sperm DNA fragmentation (SDF) in frozen semen samples of fertile and infertile groups, which showed (46.98^a^ ± 1.17; 54.42 ^a^± 1.17) in the egg-yolk + glycerol group, (36.14 ^c^± 1.20; 43.00 ^c^± 1.24) in sucrose + glycerol group and (40.93 ^b^± 1.30; 48.42 ^b^± 1.54) in the glycerol group compared to fresh semen group (30.77 ^d^± 1.29; 36.08 ^d^± 1.20) as shown in Figs. (6, 7, 8).

### Real time PCR results

**Fig. 9:**
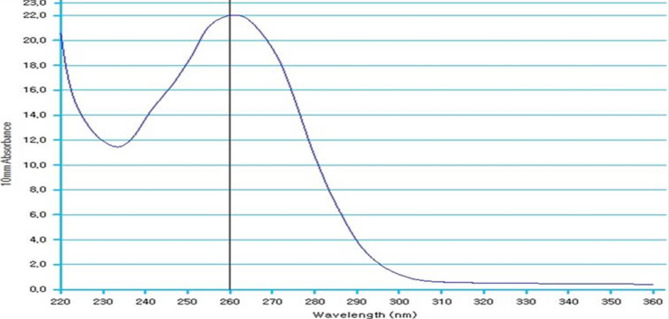
Nano-drop curve showing concentration and purity of extracted RNA from a representative sample which is 1400 ng/µl. In this curve the upper top presents at 260 and the bottom at 230 which indicates the presence of pure RNA. Relative expression of caspase-3/GAPDH gene.

The technique of real time PCR (q-PCR) in the current results detected the relative term of the apoptotic gene, caspase-3 in sperms in two groups of fertile and non-fertile samples, which refer to the changes in duplication levels of this gene after cryopreservation. To conduct real time PCR, we first isolated total RNA from semen. The concentration and goodness of RNA were detected by Nano-drop which showed presence of refined RNA. RNA, which isolated from semen samples transcribed again into c-DNA that used as a template for q-PCR. Throughout all experiment of real time PCR, *GAPDH* used as an internal reference for normalization and data was expressed as mean ± SEM (*n* = 7 in triplicate in each group). The level of the objective gene in fresh samples (G1) was considered as the base line as shown in Fig. (9).

### Effect of cryoprotective agents on relative expression of caspase-3 gene in semen samples of fertile and infertile groups

**Fig. 10:**
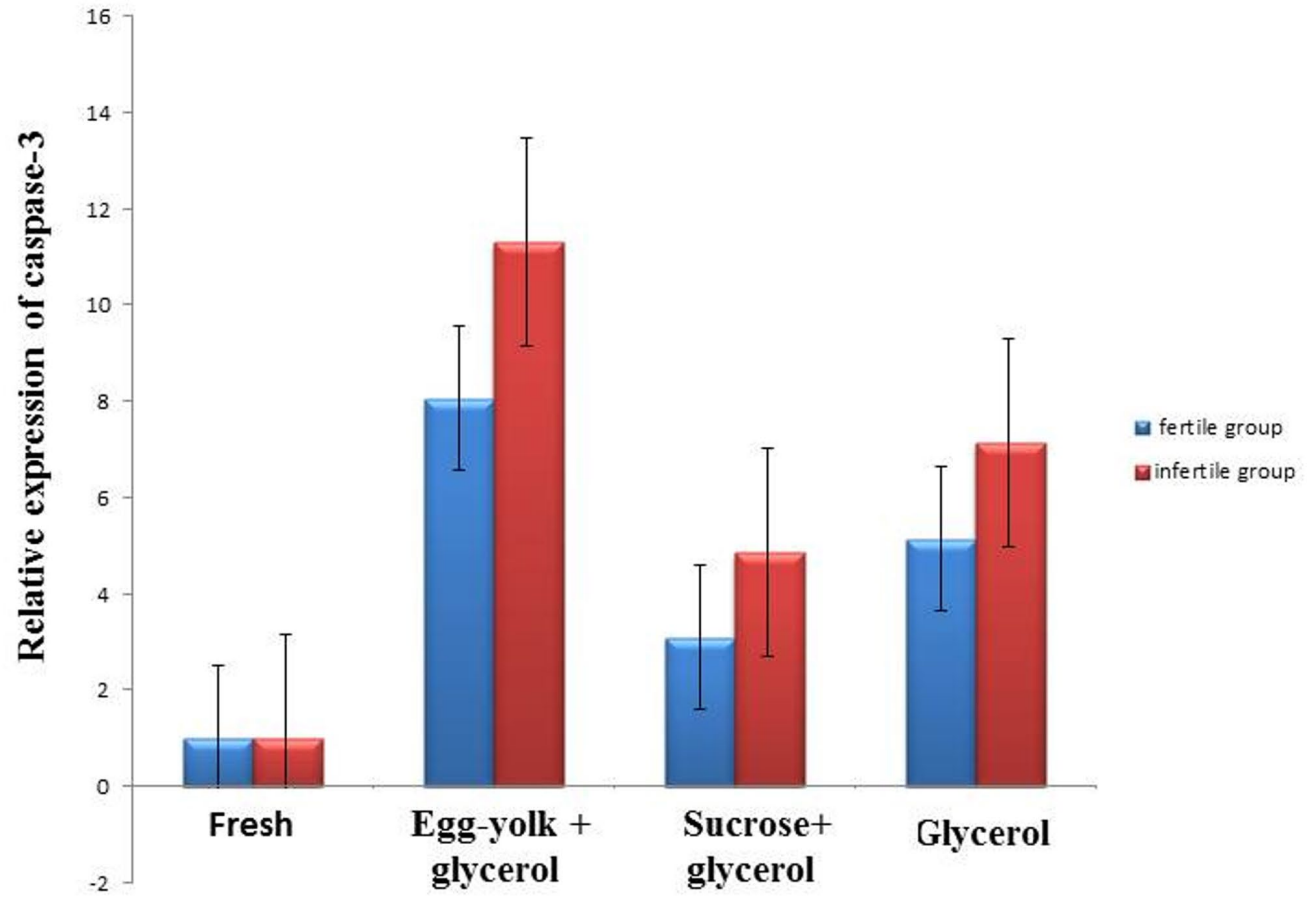
Graphical presentation of real-time quantitative PCR analysis of the expression of caspase-3 gene in semen samples of fertile and infertile groups. Data was expressed as mean ± SEM when (*n* = 15) in triplicate in each group.

The obtained results have a considerable (*P* ≤ 0.05) up-regulation in the expression of the apoptotic gene caspase-3 in fresh and frozen semen samples of fertile and infertile groups. In egg-yolk + glycerol groups (8.06 ± 0.26; 11.31 ± 0.34), sucrose + glycerol groups (3.10 ± 0.11; 4.86 ± 0.15) and glycerol groups (5.15 ± 0.19; 7.14 ± 0.28) as compared to fresh semen (1.00 ± 0.02; 1.00 ± 0.02) compared to the same cases Fig. (10).

## Discussion

Sperm cryopreservation is widely used in critical cases to preserve male fertility, particularly for patients undergoing chemotherapy, radiotherapy, cancer treatments, or surgical procedures that may damage sperm cells or the testes^5^. Compared to other cell types, sperm cells are more resistant to cryopreservation-related damage due to their low water content (approximately 50%) and the high fluidity of their cell membrane. However, despite its benefits, cryopreservation can still cause harmful alterations in sperm composition and function^6^. Current cryopreservation techniques remain imperfect, leading to significant damage to sperm membranes, reduced motility, and slower sperm movement^21^. In this study, we assessed sperm count, motility, morphology, DNA fragmentation, and apoptosis in semen samples of fertile and infertile men before and after cryopreservation.

The current results showed a decrease in sperm motility in fertile group samples compared to infertile ones. Among the different cryoprotectants tested, semen samples preserved with egg-yolk + glycerol showed the highest motility recovery after thawing, followed by sucrose + glycerol, while samples preserved with glycerol alone had the lowest post-thaw motility. Other studies have similarly reported that glycerol-based cryoprotectants alone result in significantly lower sperm motility after thawing compared to those containing egg-yolk + glycerol. The superior protective effect of egg-yolk + glycerol is attributed to its ability to better maintain sperm function compared to glycerol alone. **Hallak et al.**^22^and **Sadeghi et al.**^23^reported that a TEST-yolk buffer without glycerol resulted in higher motility recovery. This could be explained by changes in the phospholipid-to-cholesterol ratio in the sperm membrane or modifications in egg-yolk lipids, which may help preserve sperm membranes by reducing damage from free radicals. Additionally, using low concentrations of glycerol in cryoprotectants may extend the lifespan of cryopreserved sperm by minimizing osmotic damage. Sperm cells are highly sensitive to osmolality changes in their surrounding environment. When the difference in osmolality becomes too large, sperm motility decreases^24^.

In this study variations in total and progressive motility are influenced by the concentration of glycerol in cryoprotectants. Studies have shown that cryopreservation reduces progressive motility by approximately 30% in both fertile and infertile groups, with a greater decline observed in infertile individuals^25^. Progressive motility, a critical parameter for successful fertilization, reflects the spermatozoa’s capacity for forward movement. The freeze-thaw cycle associated with cryopreservation induces various forms of cellular damage, mitochondrial dysfunction, and oxidative stress, all of which contribute to the observed decline in motility. Notably, the reduction in progressive motility is more pronounced in infertile individuals, which may be attributed to the inherently reduced structural and functional integrity of their spermatozoa, rendering them more vulnerable to cryoinjury^26^.

In current findings, sperm with abnormalities showed decrease in frozen semen samples of fertile group compared to infertile individuals. Additionally, the percentage of sperm with coiled tails increased after cryopreservation. Among the different cryoprotectants used in the fertile and infertile men showed egg-yolk + glycerol was the best morphology recovery post-thaw, followed by sucrose + glycerol, while glycerol alone had the lowest protective effect. These results explained by **Hai et al.**^27^who suggested that cellular damage during freezing occurs due to membrane rupture caused by intracellular ice crystal formation during rapid freezing and extracellular ice crystal formation during slow freezing. Semen samples cryopreserved with egg-yolk + glycerol had a higher percentage of normal sperm morphology compared to those preserved with glycerol alone, making egg-yolk + glycerol the most effective cryoprotectant for the long-term storage of human sperm^28^.

In this study, frozen semen samples from the fertile group exhibited a higher level of sperm DNA fragmentation (SDF) compared to those from the infertile group. Among the cryoprotectant combinations evaluated, egg yolk with glycerol produced the highest sperm DNA fragmentation (SDF) index post-thaw, followed by glycerol alone, whereas the combination of sucrose and glycerol yielded the lowest SDF levels in both fertile and infertile groups. Similarly, **Cankut et al.**^29^ reported that cryopreserved sperm cells exhibited significant DNA fragmentation, along with membrane integrity damage and altered sperm morphology. However, some studies have found no significant relationship between sperm DNA fragmentation and head defects in sperm morphology. This may be attributed to antioxidant enzymes in seminal plasma, including urates, thiol groups, ascorbate, catalase, and superoxide dismutase (SOD), which neutralize reactive oxygen species (ROS) such as H₂O₂ and O₂. In fertile men, the total antioxidant enzyme levels in semen are higher than in infertile individuals^29^.The choice of cryoprotectant can influence sperm DNA integrity, as temperature fluctuations during cryopreservation have been shown to alter DNA structure^30^. Additionally, the sperm DNA fragmentation index (DFI) was higher in infertile men compared to fertile men, likely due to differences in DNA condensation and fertility levels. Defects in chromatin condensation and sperm structure in infertile individuals result in increased DNA instability and greater sensitivity to denaturing stress, leading to higher DNA fragmentation. Similarly, **Caliskan et al.**^31^ reported variations in sperm nuclear condensation after cryopreservation.

In the present study, Caspase-3 expression an established marker of apoptosis was assessed to further support the findings on sperm DNA fragmentation. The analysis revealed a higher proportion of Caspase-3-positive cells in frozen semen samples from the fertile group compared to those from the infertile group. Among the cryopreserved samples, those preserved with egg-yolk + glycerol exhibited the highest Caspase-3 levels post-thaw, suggesting a significant increase in apoptosis. This was followed by samples cryopreserved with glycerol alone, while sucrose + glycerol resulted in the lowest Caspase-3 levels. Once Caspase-3 is activated, apoptosis becomes irreversible making it a reliable indicator of cell death^32^. DNA fragmentation resulting from protamination failure can be detected through apoptotic markers like Caspase-3, further supporting the presence of DNA damage^15^. Previous studies have shown that fertile sperm samples are more resistant to cryopreservation-induced apoptosis compared to infertile samples^33,34^.

## Conclusion

Fertile semen samples exhibited superior post-thaw recovery in terms of motility, morphology, and DNA integrity compared to infertile samples. The slow freezing technique has proven to be the most effective method for preserving sperm motility, morphology, and DNA integrity. egg yolk combined with glycerol demonstrated the highest efficacy in maintaining motility and morphology, followed by sucrose with glycerol, while glycerol alone showed the least protective effect. The superior performance of egg yolk and sucrose is attributed to their macromolecular nature, which allows them to form a protective extracellular matrix around the sperm plasma membrane. A major limitation of the study is the absence of long-term follow-up data on the offspring derived from freeze-thawed spermatozoa with high cryo-resistance, highlighting the need for future research to evaluate potential effects on postnatal development and reproductive outcomes.

## Data Availability

Data will be available when requested from the corresponding author.
